# Enhancing detection of severe enterovirus infections: A data linkage study of ICD-10 codes with national enterovirus laboratory data, Denmark, 2010 to 2023

**DOI:** 10.2807/1560-7917.ES.2026.31.10.2500477

**Published:** 2026-03-12

**Authors:** Caroline Klint Johannesen, Amanda Marie Egeskov-Cavling, Theis Lange, Tyra Grove Krause, Micha Phill Grønholm Jepsen, Ulrikka Nygaard, Sofie E Midgley, Kristina Træholt Franck, Thea K Fischer

**Affiliations:** 1 Department of Clinical Research, North Zealand University Hospital, Hillerød, Denmark; 2 Section of Biostatistics, Department of Public Health, University of Copenhagen, Copenhagen, Denmark; 3 Epidemiological Infectious Disease Preparedness, Statens Serum Institut, Copenhagen, Denmark; 4 Department of Public Health, University of Copenhagen, Copenhagen, Denmark; 5 Department of Pulmonary and Infectious Diseases, North Zealand University Hospital, Hillerød, Denmark; 6 Department of Paediatrics and Adolescent Medicine, Copenhagen University Hospital, Rigshospitalet, Copenhagen, Denmark; 7 Faculty of Health and Medical Sciences, University of Copenhagen, Copenhagen, Denmark; 8 Department of Virology and Microbiological Preparedness, Statens Serum Institut, Copenhagen, Denmark; 9 Pandemix Center of Excellence, Roskilde University, Roskilde, Denmark

**Keywords:** Enterovirus infection, surveillance, emerging infections, database validation, viral disease, epidemiology, poliovirus

## Abstract

**INTRODUCTION:**

Enteroviruses cause symptoms ranging from mild skin manifestations to severe neurological diseases, such as polio. Despite global eradication efforts, poliovirus was detected via environmental surveillance in multiple European countries in 2024 and 2025.

**AIM:**

We aimed to assess the epidemiology of enteroviruses in Denmark, using national laboratory surveillance and hospital discharge registries, and to determine the accuracy of enterovirus registration.

**METHODS:**

Hospital admission data from 1 January 2010 to 31 December 2023 were linked to laboratory surveillance enterovirus data to identify and categorise admissions with an enterovirus-specific ICD-10 code and/or enterovirus-positive test. The accuracy of diagnosis coding and positive enterovirus tests was assessed against an estimated ‘true population’ using a capture‒recapture analysis.

**RESULTS:**

Among patients with an ICD-10 enterovirus code and a positive enterovirus test (n = 1,186), 69% had a central nervous system diagnosis. Patients with ICD-10 enterovirus codes only (n = 3,434) were younger and primarily had hand, foot and mouth disease. Patients positive for enterovirus without an enterovirus diagnosis (n = 3,263) frequently exhibited respiratory symptoms. The combined accuracy of ICD-10 codes and enterovirus tests was 46.3% (95% confidence interval (CI): 44.3–48.4) against an estimated ‘true population’ of 28,193 (95% CI: 26,929–29,457) enterovirus infections.

CONCLUSION: It is important to combine laboratory data and ICD-10 codes to gain comprehensive understanding of the enterovirus epidemiology and identify areas for improvement in enterovirus surveillance. Despite exceptional registries, the Danish system may still overlook early cases of emerging or severe enterovirus infections because of limited clinical awareness of these infections and the challenges associated with voluntary test registrations.

**Key public health message**

**What did you want to address in this study and why?**

Enterovirus infections can be asymptomatic but also cause severe neurological disease (e.g. acute flaccid myelitis) and myocarditis or pulmonary infections. We wanted to determine the precision of how enterovirus infections are registered in the data sources available for enterovirus surveillance. This allows us to address how existing data on infections can be used for surveillance and to show if clinical data are registered well enough to be used in surveillance of these viruses.

**What have we learnt from this study?**

Enterovirus infections are not always registered as such using the International Classification of Diseases-10 coding system. Some infections recorded as enterovirus infections did not have a positive test registered. Even countries with robust registries and modern laboratory facilities remain at risk of missing emerging enterovirus infections, including those with severe clinical impact such as poliovirus, and enterovirus subtypes A71 and D68.

**What are the implications of your findings for public health?**

The large gap between the two ways of registering enterovirus detections in Denmark that are used for surveillance, including for poliovirus, poses a risk for effectiveness and coverage of enterovirus surveillance at a time when (re)emerging enteroviruses are increasing. As clinical testing focuses on severe disease, using clinical testing for public health surveillance purposes is challenging and other solutions may be needed.

## Introduction

The prevalence of emerging enteroviruses (EV), including polioviruses, is increasing in Europe. In 2024 and 2025, multiple countries detected vaccine-derived poliovirus in environmental samples [1,2]. These detections were only the latest in an increasing number of outbreaks and clusters of severe diseases caused by EVs, such as subtypes A71 and D68, both of which are associated with acute flaccid myelitis (AFM) [3-11]. Clinical manifestations of enterovirus infections range from asymptomatic to severe requiring hospital admissions, with central nervous system (CNS) involvement such as meningitis or AFM, or impacts on other organ systems, such as myocarditis or pulmonary infections. With vaccination-based prevention available only for polio and EV-A71 infection and growing concerns about a possible rise of subgroups not following polio vaccination recommendations, which could facilitate virus circulation, we assessed the effectiveness of the current clinical EV surveillance to detect EV in hospitalised patients admitted with severe clinical symptoms in light of the increased concern for undetected transmission [12].

The World Health Organisation Regional Office for Europe (WHO/Europe) conducts European poliovirus surveillance through three specific surveillance systems (reports of acute flaccid paralysis (AFP), environmental surveillance, and clinical poliovirus surveillance), although not all systems are active in all countries [13]. The circulation of non-poliovirus EV in Europe is monitored by the European Network for Non-Polio Enterovirus (ENPEN) through the voluntary reporting of severe infections from laboratories and hospitals and surveillance projects on EV types of specific interest and the most common EV types detected [14]. The ENPEN has documented the presence and circulation of EVs, showing that a substantial proportion of detected infections occur in young children. The National Reference Laboratory for Polio in Denmark supports this work by providing information on EV infections and characterisation of EV subtypes, in addition to reporting poliovirus surveillance data to WHO/Europe through the clinical surveillance system. Clinical poliovirus surveillance is based on exclusion of poliovirus through typing of EV-positive samples from patients with locally diagnosed EV in any presentation, which hospitals submit to Statens Serum Institute (SSI) [15]. Together with a reporting obligation at suspicion of poliovirus infection, this passive surveillance system is so far the only way of documenting that poliovirus is not circulating in Denmark.

In this study, we assess the clinical registration of poliovirus and EV in Denmark via national EV surveillance data from the National Reference Laboratory for Polio and the Danish National Patient Register (NPR). While the burden of EV-associated hospital admissions was published in 2024 using information from the NPR, the analysis was based on ICD-10-coded admissions only [16]. Here, we aimed to enhance the understanding of severe EV infections, assess the prevalence of severe EV infections, and the accuracy of the clinical EV registration system that is used in EV surveillance. We define EV infections to be severe when they result in a contact with a Danish hospital. We hypothesised that the clinical EV registration does not cover all patients with EV. We hypothesised that positive EV tests are not always translated into diagnoses recorded in the patient’s medical file and that not all patients diagnosed with EV infections are being tested for this.

## Methods

This was a nationwide register-based data-linking study covering the period from 1 January 2010 to 31 December 2023. All inhabitants in Denmark are provided with a unique identification number (CPR number) [17]. These registration numbers are used for the identification of all contacts with health authorities and can be used to link data from multiple sources from the same person. 

### Data sources

#### National Reference Laboratory data

We included patients of all ages who tested positive for EV in a sample that had been submitted to the WHO National Reference Laboratory for Polio at SSI for surveillance. Of note, there was no registration of patients with negative or inconclusive tests. The samples were initially analysed for routine diagnostic purposes at one of the departments of clinical microbiology at Danish hospitals or at the routine diagnostic virus laboratory at SSI. Samples that tested positive for EV were submitted for surveillance on a voluntary basis. During the last 2 months of the study period, November and December 2023, the submission of EV-positive samples was mandatory if EV was detected in cerebrospinal fluid (CSF) or if the patient had symptoms indicating polio. The EV-positive clinical samples came from numerous materials. We grouped the test types as CSF, respiratory samples (sputum, saliva, tracheal matter or respiratory swabs), faecal samples (including rectal swabs) and other samples (e.g. blood, biopsy material, skin). Hospital admissions in the NPR were included for all patients with a positive EV test, admissions were investigated for relevance after data linkage.

#### National Patient Registry

In addition, we included all patients in the NPR with an enterovirus-specific diagnosis during hospital admission in the study population. Information on hospital visits for patients diagnosed with EV was obtained from the International Classification of Disease number 10 (ICD-10) diagnosis, but no information on clinical manifestations was available [18,19]. We used the following ICD-10 codes to identify EV diagnosis and co-diagnosis: DA850, DA870, DA870A, DA870B, DA80, DA801, DA802, DA803, DA804, DA809, DA880, DB303A, DB303B, DB084, DB085, DB088B, DB330, DB341, DB341A, DB341B and DB971, with further explanations in Supplementary Table S1. Hospital admissions that were ongoing at the end of the study period were excluded, as were hospital admissions related to accidents and planned follow-up visits for treatment or examination based on administrative procedure codes. We calculated the length of stay (LOS) from the start of the first registered admission date until the last timestamp of the admission. We considered two consecutive admissions as one admission if the time between discharge and the next admission was 7 days or less. Hospital admissions shorter than 12 h were classified as outpatient visits, whereas those with a minimum duration of 12 h were classified as inpatient hospital admissions. Any recurrent admissions longer than 1 h with an EV diagnosis within 90 days of discharge were examined individually to determine whether the second admission was related to the first recorded admission or whether the admission was due to a new infection requiring hospitalisation. All recurrent admissions that lasted less than 1 h were interpreted as planned follow-up visits and excluded.

#### Cause of Death Register

We used the Cause of Death Register (DAR) to identify deaths within 30 days of an EV-positive PCR test and/or the date of hospital admission. The DAR compiles information about all Danish residents who have died within Denmark [20]. The cause of death is documented in the registry as stated on the deceased person's death certificate via the ICD-10 diagnosis system. We used the system to identify natural (non-violent, not accidental) deaths during hospital admission and deaths within 30 days of admission.

### Linking of data sources

We used the Danish civil registration system, where the CPR number was used to link the data sources. Hospital admissions with and without EV-specific ICD-10 codes were linked to EV-positive tests from the national EV surveillance laboratory data. We linked a test to an admission if it was taken between 7 days before and 30 days after admission. Multiple tests could be related to the same admission. Two main groups of patients with EV infections were established: (i) patients registered in the NPR with EV-specific ICD-10 code referred to as ICD+, and (ii) patients registered with a positive EV sample in the national EV surveillance laboratory database, referred to as LAB+ . We linked these two groups, providing four subgroups: EV-coded and EV-positive (ICD+/LAB+), EV-coded and not EV-positive (ICD+/LAB−), EV-positive and an ICD code other than for EV with concurrent hospital admission (ICD−/LAB+), and EV-positive and not admitted to the hospital (LAB+/NoHosp). We did not consider admissions without an EV-specific ICD-10 code or a positive EV test relevant. We defined EV infections as severe when they resulted in a hospital admission. We investigated the mortality rate in the subgroups.

### Statistics

We analysed the data and used distribution statistics to characterise the groups and subgroups. We compared subgroups to inspect the distributions and select the methods accordingly. If the variables were dichotomous, we used chi-squared tests; if they were continuous, we used Student’s t-tests. For continuous variables that were not normally distributed, the Kruskal‒Wallis test was applied. We applied Chapmans capture–recapture framework to estimate a ‘true population’ of EV infections as a basis for validating the data through accuracy calculations. The capture‒recapture analysis followed the formula: 


$$N=\frac{(D1+1)(D2+1)\;}{a+1}-1$$

where N is the estimated ‘true population’, D1 is the number of EV infections identified only in the ICD+ group, D2 is the number of EV infections identified only in the LAB+ group, and a is the number of EV infections found in both the ICD+ and LAB+ groups. The 95% confidence interval (CI) was estimated following the same annotation with the formula: 


$$N\pm 1.96\sqrt{\frac{\left(D1+1\right)\left(D2+1\right)\left(D1-a\right)\left(D2-a\right)}{{\left(a+1\right)}^{2}\left(a+2\right)}}$$

We calculated the accuracy and corresponding 95% CI by dividing the number of EV infections in the data by the estimated ‘true population’ and the estimated confidence interval. All analyses were performed using R version 4.3.1 [21,22].

## Results

**Figure 1:**
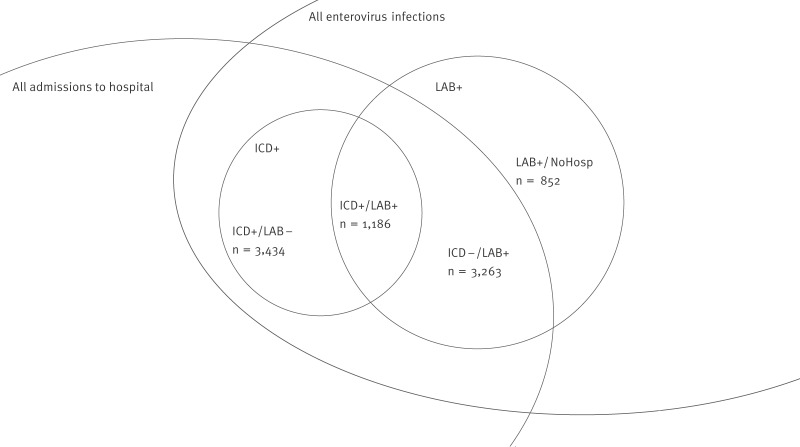
Overlap of hospitalisation data and enterovirus test data sources, with numbers of tests and admissions in the subgroups, Denmark, 2010–2023 (n = 8,735)

EV: enterovirus; ICD: International Classification of Disease; ICD +/−: EV-specific ICD-10 diagnosis; LAB +/−: national EV surveillance laboratory data; NoHosp: no related hospital admission.

We identified two main groups in the available data: the ICD+ group, consisting of 4,620 hospital admissions (2,700 inpatient admissions) with enterovirus-specific ICD-10 codes related to 4,424 unique persons, and the LAB+ group, with 7,241 EV-positive samples sent to the national surveillance laboratory, related to 6,055 unique individuals. Linking these two groups, 1,186 hospital admissions were registered with both a hospital diagnosis of EV infection and a positive EV test at the national surveillance laboratory (ICD+/LAB+), with 1,107 (93.3%) inpatient admissions (Figure 1). Information on negative EV tests was not available for these analyses. The ICD+/LAB− group had 3,434 hospital admissions (1,593 inpatient admissions; 46.4%) identified with an EV diagnosis without a positive test registered. The ICD−/LAB+ group consisted of 3,263 hospital admissions, of which 2,113 (64.8%) were inpatient admissions. Finally, in LAB+/NoHosp, 852 positive tests were not related to hospital admissions. The characteristics of the two main groups are described below, and the four subgroups are compared in Table 1.

**Table 1:** Characteristics of subgroups with enterovirus diagnosis and/or positive laboratory, Denmark, 2010–2023 (n = 8,735)

Group	All groups		ICD+/LAB+		ICD+/LAB−		ICD−/LAB+		LAB+/NoHosp		p value^a^
	n	%	n	%	n	%	n	%	n	%	
**Total**	**8,735**	**100**	**1,186**	**100**	**3,434**	**100**	**3,263**	**100**	**852**	**100**	**NA**
Median age in years (IQR)	1.0 (0–22)		19.0 (0–32)		1.0 (1–15)		1.0 (0–4)		19.5 (1–33)		< 0.001
Age < 18 years	6,301	72.1	583	49.2	2,596	75.6	2,710	83.1	412	48.4	< 0.001
Sex female	3,730	42.7	518	43.7	1,458	42.5	1,372	42.0	382	44.8	0.44
Sex male	5,005	57.3	668	56.3	1,976	57.5	1,891	58.0	470	55.2	
Admission type inpatient	4,813	55.1	1,107	93.3	1,593	46.4	2,113	64.8	NA	NA	< 0.001
Median LOS for inpatients (IQR)	2 (1 –4)		2 (1–4)		2 (1–4)		1 (1–3)		NA	NA	< 0.001
Mortality 30 days^b^	45	0.5	0	0	7	0.2	30	0.9	8	0.9	< 0.001

EV: enterovirus; ICD: International Classification of Disease; ICD+/−: EV-specific ICD-10 diagnosis; IQR: interquartile range; LAB +/−: national EV surveillance laboratory data; LOS: length of stay, measured in days; NA: not applicable; NoHosp: no related hospital admission.

^a^ p value for test of null hypothesis of equal distributions in the subgroups.

^b^ Natural cause mortality within each subgroup, deceased up to 30 days after admission date or test date.

### ICD+: enterovirus-specific ICD-10-coded admissions

The 4,620 EV-coded admissions were predominantly registered with ICD-10 codes related to infections (n = 4,034; 87%) and CNS (n = 1,539; 33%), and 1,859 (40%) had diagnoses related to infections in the skin, with enterovirus as their primary cause of admission (Table 2). Patient ages ranged from 0 to 96 years, with a median age of 2 years (interquartile range (IQR): 2–26), where 69% (n = 3179) of the patients were younger than 18 years, and the majority (57%) were male (2,644 male and 1,976 female. 

**Table 2:** Number and proportions of enterovirus admissions according to ICD-10 diagnosis codes, Denmark, 2010–2023 (n = 7,883^a^)

Diagnosis group	ICD+				ICD+/LAB+				ICD+/LAB−				ICD−/LAB+				p value ^b^
	All		Inpatient		All		Inpatient		All		Inpatient		All		Inpatient		
	n	%	n	%	n	%	n	%	n	%	n	%	n	%	n	%	
**Total**	**4,620**	**100**	**2,700**	**100**	**1,186**	**100**	**1,107**	**100**	**3,434**	**100**	**1,593**	**100**	**3,263**	**100**	**2,113**	**100**	** NA**
Admissions with positive EV tests	1,186	25.7	1,107	41	1,186	100	1,107	100	0	0	0	0	3,263	100	2,113	100	NA
ICD-10:A80-A87, A881, A888, A89, G00-G03, G05, G09, R26, R29, Central nervous system diseases																	
CNS diagnosis	1,539	33.3	1,425	52.8	856	72.2	834	75.3	683	19.9	591	37.1	368	11.3	356	16.8	< 0.001
ICD-10:A00-B99, Certain infectious and parasitic diseases																	
Infections, including CNS infections	4,034	87.3	2,231	82.6	1,063	89.6	933	84.3	2,971	86.5	1,298	81.5	793	24.3	539	25.5	< 0.001
ICD-10: C00-C99, D0-D4, Cancers, malignant neoplasms and in situ neoplasms																	
Cancer	25	0.5	12	0.4	5	0.4	< 5	NA	20	0.6	8	0.5	79	2.4	48	2.3	< 0.001
ICD-10: K00-K93, Diseases of the digestive system																	
Gastro-intestinal disease	20	0.4	15	0.6	< 5	NA	< 5	NA	19	0.6	< 15	NA	17	0.5	11	0.5	0.107
ICD-10: D50-D89, Diseases of the blood and blood-forming organs and certain disorders involving the immune mechanism																	
Disease of the blood and immune system	13	0.3	< 10	NA	< 5	NA	< 5	NA	11	0.3	< 10	NA	22	0.7	13	0.6	0.028
ICD-10: J00-J99, Diseases of the respiratory system																	
Pulmonary disease	150	3.2	114	4.2	29	2.4	< 25	NA	121	3.5	88	5.5	1,446	44.3	840	39.8	< 0.001
ICD-10: I00-I99, Diseases of the circulatory system																	
Cardiovascular diseases	21	0.5	< 15	NA	6	0.5	6	0.5	15	0.4	< 10	NA	57	1.7	42	2	< 0.001
ICD-0: E00-E90, Endocrine, nutritional and metabolic diseases																	
Endocrine	54	1.2	48	1.8	13	1.1	13	1.2	41	1.2	35	2.2	56	1.7	40	1.9	0.121
ICD-10: F00-F99, Mental and behavioural disorders																	
Psychiatric	11	0.2	0	0	0	0.0	0	0	11	0.3	0	0	9	0.3	< 5	NA	0.159
ICD-10: G00-G99, Diseases of the nervous system																	
Nervous system	256	5.5	251	9.3	149	12.6	144	13	107	3.1	107	6.7	304	9.3	272	12.9	< 0.001
ICD-10: M00-M99, Diseases of the musculoskeletal system and connective tissue																	
Bones and muscles	22	0.5	15	0.6	< 5	NA	< 5	NA	19	0.6	13	0.8	< 5	NA	< 5	NA	0.008
ICD-10: L00-L99, Diseases of the skin and subcutaneous tissue																	
Skin disease	65	1.4	33	1.2	< 5	NA	< 5	NA	62	1.8	31	1.9	30	0.9	17	0.8	< 0.001
ICD-10: P00-P96, Certain conditions originating in the perinatal period																	
Perinatal disease	24	0.5	< 20	NA	9	0.8	9	0.8	15	0.4	< 10	NA	20	0.6	14	0.7	0.378

CNS: central nervous system; EV: enterovirus; ICD: International Classification of Disease; ICD +/−: EV-specific ICD-10 diagnosis; LAB +/−: national EV surveillance laboratory data; NA: not applicable; NoHosp: no related hospital admission.

^a^ This table excludes the NoHosp subgroup.

^b^ p value for comparisons of ICD+/LAB+, ICD+/LAB− and ICD−/LAB+ (all admissions).

Distribution of registered primary diagnoses among admissions with enterovirus infections and inpatient admissions (≥ 12 h) in ICD+, ICD+/LAB+, ICD+/LAB− and ICD−/LAB+ (multiple codes per admission possible).

**Figure 2:**
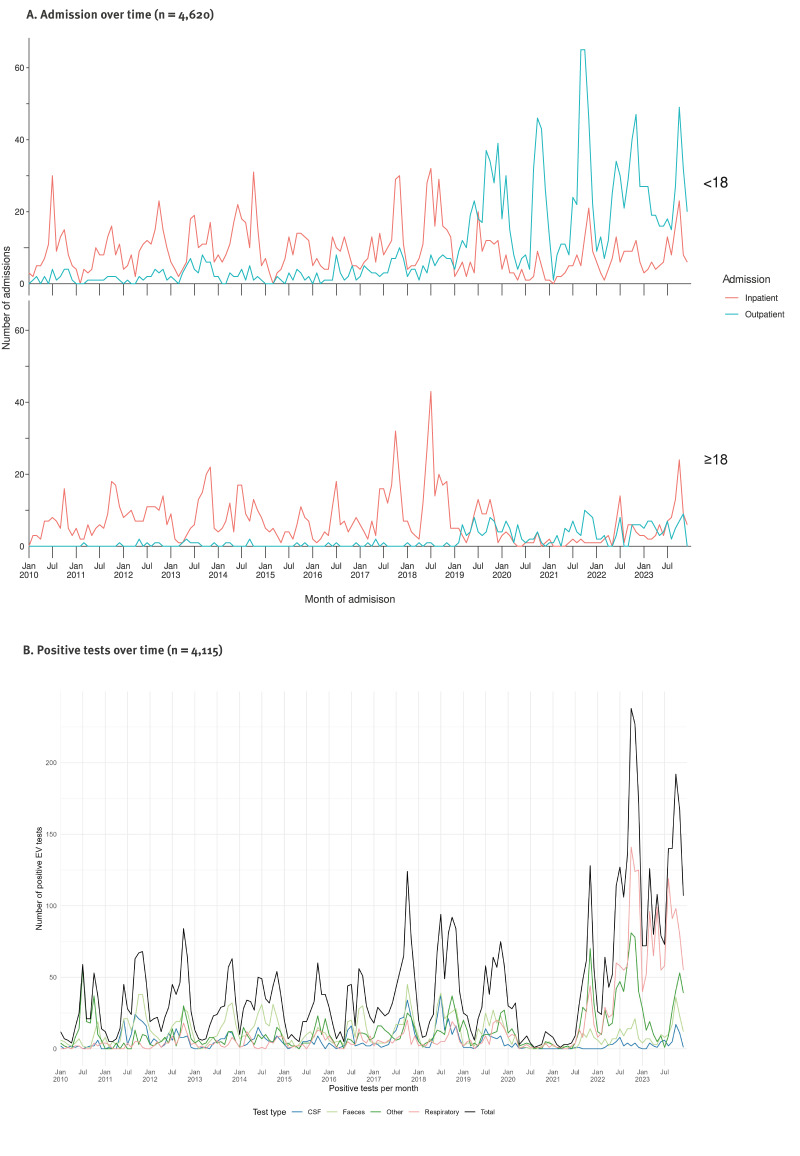
Number of admissions with enterovirus-specific ICD-10 diagnosis per month by age group, patient type based on of admission and length^a^ and number of enterovirus-positive per month by test type, Denmark 2010–2023 (n = 8,735)

CSF: cerebrospinal fluid; EV: enterovirus; ICD: International Classification of Disease.

^a^ Admission type in-/outpatient is based on length of stay ≥ 12 h / < 12 h.

The number of hospitalisations increased during the studied years (Figure 2). The majority of admissions were inpatient admissions (n = 2,700; 58%), although outpatient visits dominated from 2019 onwards. The changes in admission type over time were most predominant among patients younger than 18 years (Figure 2).

Panel B:Test types are grouped as CSF, respiratory samples (sputum, saliva, tracheal matter or respiratory swabs), faecal samples (including rectal swabs) and other samples (e.g. blood, biopsy material, skin).

### LAB+: positive enterovirus-specific test

The number of positive EV tests also increased during the studied years. The increase was especially large for respiratory samples, which were primarily collected between 2022 and 2023, although a general decline in positive samples characterised the COVID-19 pandemic period from 2020 to 2022. Each year, the monthly pattern of positive tests showed seasonal peaks in the northern hemisphere summer and autumn months, except in 2020, when nearly no positive tests were recorded (Figure 2). The age of the patients at the time of sample collection ranged from 0 to 99 years, with a median age of 2 years (IQR: 0–27). The mean age was 13.1 years, indicating a left-skewed age distribution in this group. Similar to the ICD+ group, 69% of the patients were younger than 18 years, and the majority were male (57% male, 43% female).

### Comparison of subgroups

The distribution of inpatient and outpatient hospital contacts and the median age of the patients differed between the subgroups, with ICD+/LAB+ as an outlier with a median age of 19 years (Table 1). Inpatient admissions were most common in the ICD+/LAB+ group, where they constituted 93% of the admissions. The sex distribution was equal across the four subgroups.

In ICD+/LAB+, ICD+/LAB− and ICD−/LAB+ patients, we examined the distribution of large diagnostic groups of diagnoses , to describe and understand how admissions with enterovirus present clinically (Table 2). The large diagnostic groups follow the main chapters of the ICD-10 system, plus a CNS infections group. There were significant differences in the diagnostic distribution between ICD+ LAB+, ICD+/LAB− and ICD−/LAB+, except for psychiatric diagnoses, gastro-intestinal disease, endocrine, nutritional and metabolic diseases, and diagnoses related to the perinatal period. In the ICD+/LAB+ group, admissions were often CNS infections, whereas in the ICD−/LAB+ group, admissions with pulmonary diseases were the most common. The ICD−/LAB+ group had the lowest proportion of admissions with codes related to infections.

**Figure 3:**
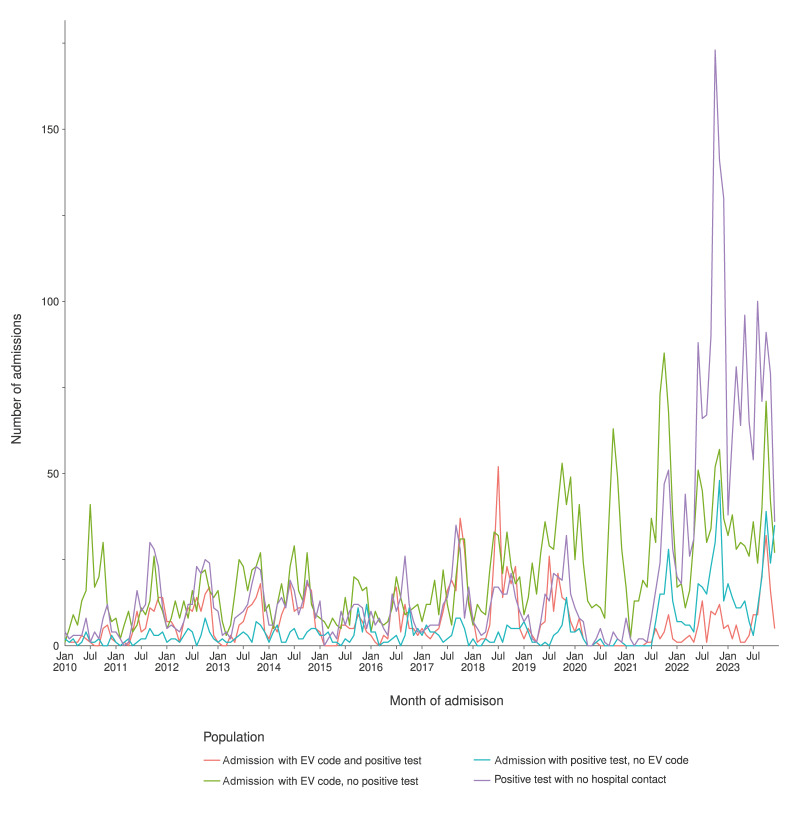
Number of enterovirus admissions per month in the subgroups ICD+/LAB+, ICD+/LAB−, ICD−/LAB+ and LAB+/NoHosp groups, Denmark, 2010–2023 (n = 8,735)

EV: enterovirus; ICD: International Classification of Disease; ICD +/−: EV-specific ICD-10 diagnosis; LAB +/−: national EV surveillance laboratory data; NoHosp: no related hospital admission.

At the beginning of the study period, the number of admissions per month for ICD+/LAB+, ICD+/LAB− and ICD−/LAB+ was relatively stable. However, from 2019 onwards, the trends began to diverge. The number of ICD+/LAB+ admissions declined, whereas the number of ICD+/LAB− and ICD−/LAB+ admissions increased (Figure 3).

**Figure 4:**
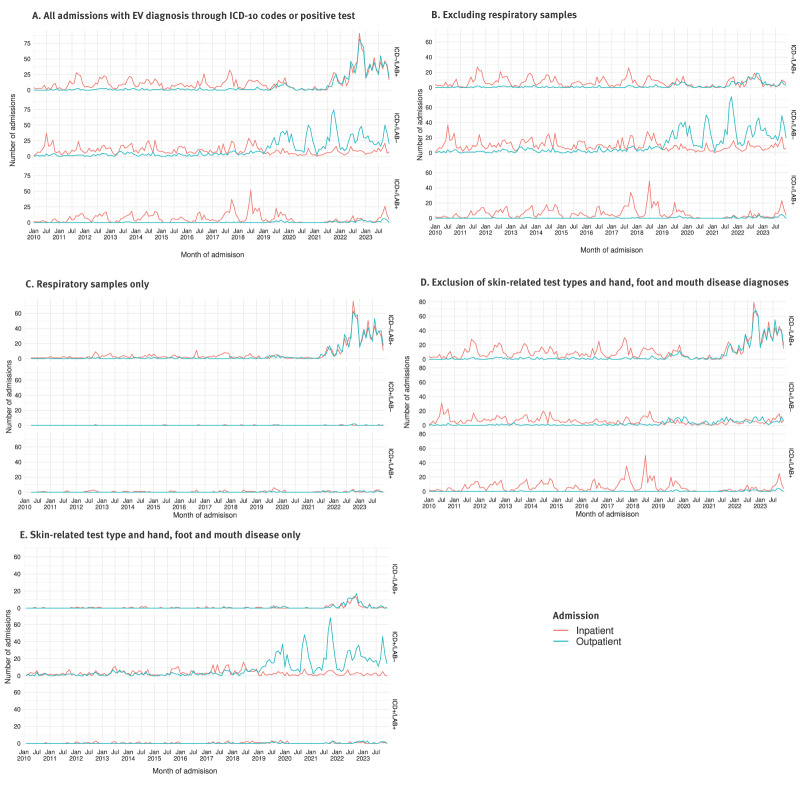
Number of admissions per month and admission type in the subgroups ICD+/LAB+, ICD+/LAB−, and ICD−/LAB+, Denmark 2010–2023 (n = 8,735)

EV: enterovirus; ICD: International Classification of Disease; ICD +/−: EV-specific ICD-10 diagnosis; LAB +/−: national EV surveillance laboratory data.

We analysed this pattern further by categorising admissions into inpatient and outpatient admissions based on the length of stay. In the ICD+/LAB− group, the increase in admissions was primarily due to outpatient cases. In contrast, in the ICD−/LAB+ group, the increase was equal for both inpatient and outpatient admissions (Figure 4A).

When we restricted the admissions included in the analysis, the increase in ICD−/LAB+ admissions was related almost exclusively to respiratory samples, while those contributed very few admissions to ICD+/LAB+ and ICD+/LAB− admissions (Figure 4, panels B and C). The increase in admissions in ICD+/LAB− outpatient admissions was related to samples from skin and hand, foot and mouth disease (HFMD) diagnostic codes (Figure 4, panels D and E).

### Estimates from capture‒recapture analyses and accuracy of detection

Using the capture–recapture method, the ‘true population’ of EV infections was 28,193 cases (95% CI: 26,929–29,457). This translates to an accuracy of ICD+ alone of 20.6% (95% CI: 19.7–28.8). The accuracy of LAB+ alone was 29.9% (95% CI: 28.6–31.3). The combined accuracy of the two systems was 44.3% (95% CI: 44.3–48.4).

## Discussion

This nationwide register-based data-linking study combined data from the national enterovirus surveillance database and the hospital admissions in NPR to assess the use of EV-specific ICD-10-codes and positive EV test results in clinical registration and surveillance of EV infections and to present the epidemiology of EV infections. There was very little overlap between the group of hospitalised patients who had received an ICD-10 diagnosis of EV and the group of patients who had tested PCR-positive for EV. Thus, the majority of hospitalised patients who tested PCR-positive for EV were not registered with an ICD-10 code for EV. Only a quarter of patients who were registered with an ICD-10 code for EV were also registered with a positive EV PCR test, forming the subgroup ICD+/LAB+. Admissions in ICD+/LAB+ were characterised by older age, inpatient stays, infections in general and CNS infections in particular.

The majority of patients with a clinical diagnosis of EV without a positive laboratory result (ICD+/LAB−) were diagnosed with HFMD. This supports our hypothesis that patients with EV-specific diagnoses do not always undergo concomitant microbiology tests, especially when the symptoms are easily distinguishable, as in the case of HFMD. The positive tests that did not have EV-specific diagnoses (ICD−/LAB+, LAB+/NoHosp) were dominated by respiratory tests and admissions for diseases related to the pulmonary system. While the rise in respiratory testing following the COVID-19 pandemic led to an expected increase in EV-positive respiratory tests, the corresponding admissions were not registered as EV infections in the NPR.

Patients with positive EV tests without related admissions (LAB+/NoHosp) had a demographic structure similar to ICD+/LAB+, but a mortality rate within 30 days of 9 per 1,000 positive tests compared with 0 in ICD+/LAB+. This suggests that additional focus could be placed on these patients to investigate whether hospital admission could prevent some of these deaths.

When we calculated the accuracy of the EV-specific ICD-10 codes (ICD+) and positive EV tests (LAB+) compared with the estimated ‘true population’ from the capture–recapture analysis, it was clear that neither the ICD+ nor the LAB+ accurately captured the full span of EV infections. With a combined accuracy of 46%, the databases did not capture the majority of the ‘true positives’. However, the two databases included capture mostly severe EV infections and infections with a recognisable symptom (ICD+) and tests taken for clinical diagnostics (LAB+), all of which require medical attendance to the infection. And the ‘true positive’ estimation holds no assumption of the severity of infections included in the ‘true population’ which will also include non-severe and subclinical EV infections. Given this heterogeneity in registration probability, the estimate of the ‘true population’ should be interpreted with caution. Analysis of bias from similar situations shows a tendency for underestimation of the true population, which may include even more non-severe and subclinical infections.

Seroprevalence studies confirm that the majority of children will have had an EV infection. Just two EVs (EVA71 and CVA6), which cause HFMD, reach seroprevalences upwards of 75% of the tested at age 10 years [23]. While no other study has validated the use of EV-specific ICD-10 codes in NPR and positive tests, Egeskov-Cavling et al. validated the specific ICD-10 codes for respiratory syncytial virus (RSV) among Danish adults via the NPR and tests from the national database of microbiology (MiBa) and reported that 57% of adults with positive RSV tests who were admitted to the hospital with a respiratory infection did not have a specific RSV diagnosis [24]. In EV infections, coding and tests seem to coincide more often in severe CNS infections. Jørgensen et al. documented a high sensitivity of the ICD-10 codes for herpes encephalitis using the same data sources as Egeskov-Cavling. Based on these studies, it can be assumed that the accuracy, especially combining the two databases, increases with the severity of the disease [25], while differences in clinical awareness and the absence of disease-specific treatment may further influence the completeness of registration. 

The present study had several strengths, including full population coverage, 14 years of data, and the inherent robustness of the NPR, which provides comprehensive information on all hospital admissions in Denmark. The ability to link national EV laboratory surveillance data to the NPR enabled not only the study of the aggregated number of identified patients but also a detailed description of the subgroups where the two systems overlapped and where they did not.

However, there are limitations, primarily with the national EV laboratory surveillance data. The database only contains positive samples and, owing to the voluntary submission of EV-positive samples, it cannot be expected to hold all positive samples. Condell et al. evaluated the flow of EV tests submitted to the Danish national database between 2010 and 2013. They recommended improving the reporting system for EV to contribute to this database based on increased awareness of the risk of imported polio cases [15]. Following Condell et al.’s evaluation, there was an increase in reported positive test results in subsequent years. This increase may be linked to the recommendations of the evaluation and increased awareness, although it is more likely explained by advances in broader syndromic testing at local laboratories. Without information on negative tests, we could not exclude patients who had an EV diagnosis that was disconfirmed by a negative test after the end of admission. In the ICD+/LAB− codes, we did find a proportion of CNS diagnoses (32% of inpatient admissions), indicating a probable inconsistency in the laboratory surveillance data, as pathogen-specific diagnoses of CNS infections are rare without microbiological testing. Without access to patients' healthcare records, it was not possible to investigate if tests had been conducted but were missing from the surveillance database, or if tests had been negative for EV but the results had not altered the ICD-10 diagnostics. 

The results from our use of EV testing data to validate the use of EV-related ICD-10 codes in hospital admissions support the hypothesis that easily distinguishable diseases, such as HFMD, are often exempt from testing. It also showed that a clinical diagnosis of EV infection coincides with a test for EV only if the patient is severely affected, as with CNS infection. In skin-related and pulmonary disease, the diagnostic codes and tests were not jointly present. While the combined accuracy of the databases is fair, they do seem best at describing severe infections. If only patients with severe disease are tested for EV and registered with a diagnosis accordingly, there is a risk that viral transmissions can go undetected and that viruses with the potential to cause severe outbreaks can spread and evolve undetected. Thus, ensuring that polioviruses and other EVs are always detected poses a challenge. As clinical testing focuses on severe disease, using clinical testing for public health surveillance purposes is challenging and other solutions may be needed. Wastewater-based environmental surveillance of EV could be considered a measure to increase detection sensitivity, especially with regard to EV types not yet causing severe diseases. To enhance the use of clinical and test data for surveillance purposes, the mandatory submission of EV-positive CSF samples could be expanded to all EV-positive samples from hospitalised patients, along with a registration of the non-EV-positive samples for better clinical EV surveillance in the future. A surveillance tool that will enable the surveillance of all samples that are tested for EV, whether positive or negative, could be the next step in Denmark.

## Conclusion

This study illustrates how a country with robust registries and modern laboratory facilities remains at risk of missing emerging EV infections, including those with severe clinical impact. These findings further translate to challenges in the current poliovirus surveillance systems in many countries, as well as potential future EV surveillance. Moreover, it highlights the importance of combining diagnostic laboratory data with hospital-level ICD-10 codes when assessing the prevalence of severe EV disease in a country.

## Data Availability

The datasets presented in this article are not readily available due to Danish data protection legislation. All register data in Denmark is accessible for researchers with approval from the relevant authorities. Requests to access the datasets should be directed to The Danish Health Data Authority (Sundhedsdatastyrelsen).

## Supplementary Data

185000 Supplement
