# Supplementary material for: Enhancing detection of severe enterovirus infections: A data linkage study of ICD-10 codes with national enterovirus laboratory data, Denmark, 2010 to 2023
Source: Euro Surveill. 2026 Mar 12;31(10):2500477. doi: 10.2807/1560-7917.ES.2026.31.10.2500477 (PMC13074322; doi:10.2807/1560-7917.ES.2026.31.10.2500477)
Supplement: Supplement [file 25-00477_JOHANNESEN_Supplement.pdf]

Supplementary material

This supplementary material is hosted by Eurosurveillance as supporting information alongside the article [Enhancing detection of severe enterovirus infections: A data linkage study of ICD-10 codes with national enterovirus laboratory data, Denmark, 2010 to 2023], on behalf of the authors, who remain responsible for the accuracy and appropriateness of the content. The same standards for ethics, copyright, attributions and permissions as for the article apply. Supplements are not edited by Eurosurveillance and the journal is not responsible for the maintenance of any links or email addresses provided therein.

Supplementary Table S1: ICD-10 codes used for the EV-admission and co-diagnosis groups.

ICD-10 code	Diagnostic definition	Diagnostic group Notes
DA850	Enteroviral encephalitis	CNS-disease
DA870, DA870A, DA870B	Enteroviral meningitis	CNS-disease<br>A: Coxsackievirus,<br>B: Echovirus
DA80	Acute poliomyelitis	CNS-disease
DA801	Acute paralytic poliomyelitis, wild virus, imported	CNS-disease
DA802	Acute paralytic poliomyelitis, wild virus, indigenous	CNS-disease
DA803	Acute paralytic poliomyelitis, other and unspecified	CNS-disease
DA804	Acute nonparalytic poliomyelitis	CNS-disease
DA809	Acute poliomyelitis, unspecified	CNS-disease
DA880	Enteroviral exanthematous fever [Boston exanthem]	Skin-disease
DB303A, DB303B	Acute epidemic hemorrhagic conjunctivitis (enteroviral)	Other organ system<br>A: Coxsackievirus type 24,<br>B: Enterovirus type 70
DB084	Enteroviral vesicular stomatitis with exanthem	Skin-disease<br>Hand-, foot-, and mouth-disease (HFMD)
DB085	Enteroviral vesicular pharyngitis	Skin-disease
DB088B	Enteroviral lymphonodular pharyngitis	Skin-disease
DB330	Bornholm disease	Other organ system
DB341, DB341A, DB341B	Enterovirus infection, unspecified site	Other organ system A: Coxsackievirus,<br>B: Echovirus
DB971	Enterovirus as the cause of diseases classified to other chapters	Other organ system

ICD-10 codes	Co-diagnosis group
DA80, DA81, DA82, DA83, DA84, DA85, DA86, DA87, DA881, DA888, DA89, DG00, DG01, DG02, DG03, DG05, DG09, DR26, DR29	CNS disease

DA850, DA870, A870A, DA870B, DA80, DA801, DA802, DA803, DA804, DA809	CNS enterovirus-specific disease
DA00-DB99	Infection
DC00-DC99, DD0-DD4	Cancer
DK00-DK93	Gastro-intestenal disease
DD50-DD89	Disease of blood and immune system
DJ00-DJ99	Pulmonary disease
DI00-DI99	Cardiovascular disease
DE00-DE90	Endocrine disease
DF00-DF99	Psychiatric disease
DG00-DG99	Nervous system disease
DM00-DM99	Bones and muscles diseases
DL00-DL99	Skin disease
DP00-DP96	Perinatal states and disease

*CNS: Central Nervours system
